# The Complex Balance between Analgesic Efficacy, Change of Dose and Safety Profile Over Time, in Cancer Patients Treated with Opioids: Providing the Clinicians with an Evaluation Tool

**DOI:** 10.3390/jcm9020502

**Published:** 2020-02-12

**Authors:** Oscar Corli, Luca Porcu, Claudia Santucci, Cristina Bosetti

**Affiliations:** 1 Department of Oncology, Laboratory of Methodology for Clinical Research, Unit of Pain and Palliative Care Research, Istituto di Ricerche Farmacologiche Mario Negri IRCCS, 20156 Milan, Italy; 2 Department of Oncology, Laboratory of Methodology for Clinical Research, Unit of Methodological Research, Istituto di Ricerche Farmacologiche Mario Negri IRCCS, 20156 Milan, Italy; luca.porcu@marionegri.it; 3 Department of Oncology, Laboratory of Methodology for Clinical Research, Unit of Cancer Epidemiology, Istituto di Ricerche Farmacologiche Mario Negri IRCCS, 20156 Milan, Italy; claudia.santucci@marionegri.it (C.S.); cristina.bosetti@marionegri.it (C.B.)

**Keywords:** analgesic response, cancer, dose escalation, opioids, safety

## Abstract

Background: Scanty data exist on the integration between the analgesic effect of opioids, dose changes, and adverse events in cancer patients. Methods: To provide further information on this issue, we analysed data on 498 advanced-stage cancer patients treated with strong opioids. At baseline and three visits (at days 7, 14, and 21), pain intensity, oral morphine-equivalent daily dose, and the prevalence of major adverse events were measured. The proportion of responders (pain intensity decrease ≥30% from baseline) and non-responders, as well as of patients with low or high dose escalation, was calculated. Results: Pain intensity strongly decreased from baseline (pain intensity difference −4.0 at day 7 and −4.2 at day 21) in responders, while it was quite stable in non-responders (pain intensity difference −0.8 at day 7 and −0.9 at day 21). In low dose escalation patients (82.4% at final visit), daily dose changed from 52.3 to 65.3 mg; in high dose escalation patients (17.6%), it varied from 94.1 to 146.7 mg. Among responders, high dose escalation patients experienced significantly more frequent adverse events compared to low or high dose escalation patients, while no differences were observed in non-responders. Conclusions: The response to opioids results from the combination of three clinical aspects, which are strongly interrelated. These results provide some thoughts to help clinical evaluations and therapeutic decisions regarding opioid use.

## 1. Introduction

Opioids are considered the most effective drugs to relieve severe cancer pain, as indicated by several guidelines and recommendations [1,2,3]. The opioid treatment over time, however, cannot only be evaluated on the basis of the analgesic effect but should also consider the dose necessary to obtain and maintain pain reduction and a safety profile.

In general, analgesia is not a constant outcome but tends to vary among patients and time [4,5]. Given a pain reduction by at least 30% as a cut-off for satisfactory clinical results [6], good pain control has been reported in 50%–90% of cancer patients [7,8]. Non-responsiveness is not a rare condition and, up to now, it has only been partially investigated in the literature. Cancer pain is a singular clinical entity, defined by multimorphic characteristics, that can be modified during cancer progression by various factors. These include both cancer etiology, physiopathology, and clinical presentation, as well as disruptive elements, as concomitant treatments, pain from associated diseases, comorbidities and complications, and modifications in the environment [9]. Furthermore, the presence of neuropathic and breakthrough pain worsens pain intensity and induces a poorer response to analgesics [10,11].

Personalised, interventional, and multimodal management, aiming to give an exhaustive approach to all factors influencing pain, must be carefully considered. Moreover, facing poor analgesia, the clinician tends to apply a compensatory increase in the opioid dose. This choice may give a temporary benefit that often disappears over time. Indeed, prolonged and continuous use of opioids, with a compensatory increase of dose, produces two types of neuroadaptation that interfere with opioid ability to provide analgesia [12]. The first neuroadaptation is tolerance, which is characterised by a progressive lack of response due to an adaptive mechanism that neutralises the drug effect by the opioid receptors desensitisation [13,14]. The second neuroadaptation is known as opioid-induced hyperalgesia, where opioids, paradoxically, cause pain hypersensitivity [15]. Hyperalgesia is a rare complication, dose dependent, mediated by the activation of specific pronociceptive processes, generally outside the opioidergic system [16,17]. Consequently, there is a need to establish a proper titration of opioids, aiming to achieve a defined optimal dose that could balance the benefits and harms of opioids in managing cancer pain. Important clinical parameters of adverse events (AEs) are prevalence, severity, and changes over time. The balance between opioid benefits and harms is fundamental in deciding the best management of pain in cancer patients. 

We carried out this analysis with the aim of evaluating the analgesic responses in advanced-stage cancer patients treated with strong opioids over a period of treatment of three weeks and describe the course of the treatment in terms of analgesic effect, required doses, and AEs. 

## 2. Material and Methods

This is a post-hoc analysis from a randomised, open-label, longitudinal, phase IV clinical trial [18,19] on advanced-stage cancer patients experiencing moderate to severe pain, randomised to receive one WHO Step III opioid (oral morphine, oxycodone, transdermal fentanyl, or buprenorphine), never administered previously. In this context, we considered patients allocated to the four opioids as a single group of patients. 

The eligibility criteria and study details are described in the original study publication [18]. Briefly, the initial opioid doses were based on the recommendations of the European Association for Palliative Care [20], starting with 30 to 60 mg daily of oral morphine-equivalent daily dose (OMEDD), in respect of patient’s general clinical condition, age, and previous analgesic therapy. During the follow-up, physicians (oncologists and palliative care or pain therapists) were allowed to modify doses, change the opioid, or discontinue the treatment, based on their experience and patient’s clinical needs.

The study consisted of six visits, including the baseline visit and five follow-up visits at days 3, 7, 14, 21, and 28. At each visit, pain intensity (PI) was assessed as average pain intensity (API) experienced by the patient in the previous 24 hours by means of a numeric rating scale, from 0 (no pain) to 10 (worst imaginable pain). Moreover, at each visit, the prescribed opioid daily dose, expressed in OMEDD, was recorded. Moreover, the main opioid-induced AEs were also assessed using the Therapy Impact Questionnaire [21], where patients self-reported the presence and degree of six most frequent AEs (i.e., confusion, constipation, drowsiness, dry mouth, nausea, and vomiting) experienced over the previous week. 

For this analysis, only the baseline visit and those at days 7, 14, and 21 were considered since we aimed to evaluate the role of the administered opioids with the associated dosages after one week of treatment. The visit at day 28 was not considered since 166 patients dropped-out for different reasons (36 of them, 22%, died). Pain intensity difference (PID) between baseline and each subsequent visit was calculated and used to classify patients as responders (i.e., ≥30% pain intensity reduction) or non-responders (i.e., <30% decrease in pain intensity), based on Farrar’s criteria [6]. The Opioid Escalation Index (OEI) was calculated [22], and patients were then grouped into high dose escalation; i.e., OEI >5% respect to initial dose) and low dose escalation (i.e., OEI ≤ 5%).

Original study approval was obtained by the review boards of each centre, and patients gave their written informed consent.

### Statistical Analysis

Mean and standard deviation (SD) and absolute and percentage frequencies were used to describe continuous and categorical variables, respectively. Trends in API, PID, mean dose, and high dose escalation over time (from days 7 to day 21) were tested using linear regression models. Moreover, differences across responders and non-responders were evaluated by independent samples *t*-test. The profile of safety was analysed according to the combination of analgesic response (responders and non-responders) and dose escalation (low dose escalation and high dose escalation), and logistic regression models were used to estimate the odds ratio (OR) of AEs for high dose escalation patients as compared to low dose escalation patients, among responders and non-responders. The Breslow–Day test was used to detect heterogeneity of OR estimates between responders and non-responders. All the analyses were carried out with the SAS Software, version 9.4 (SAS Institute, Cary, North Carolina, USA).

## 3. Results

Table 1 shows the baseline demographic and clinical characteristics for the patients included in the study.

Table 2 reports API and PID at baseline and at days 7, 14, and 21, overall, and according to the analgesic response. Among all patients, overall, the API score decreased over time (from 6.0 at baseline to 2.4 at day 21), the API score decreased in the responders (from 2.1 to 1.9), while it was stable in non-responders (5.0 and 4.9). Corresponding changes in PID were from −3.1 at day 7 to −3.6 at day 21 in all patients (*p*-value for trend: 0.002), from −4.0 to −4.2 in responders (*p*-value for trend: 0.063), and from −0.8 to −0.9 in non-responders (*p*-value for trend: 0.575).

Table 3 shows the distribution of low dose escalation and high dose escalation patients and their respective opioid daily doses over time. Opioid daily dose increased over time, overall (from 63.4 mg at day 7 to 79.6 mg at day 21; *p*-value for trend <0.001) and according to dose escalation status (from 52.3 to 65.3 mg in low dose escalation patients, *p*-value for trend <0.001; and from 94.1 to 146.7 mg in high dose escalation patients, *p*-value for trend <0.001; *p*-value: < 0.001 for the difference between low dose escalation and high dose escalation at day 21).

Mean dose and the prevalence of high dose escalation patients according to analgesic response (responders or non-responders) are reported in Table 4. Mean doses were higher in non-responders than responders at each visit (day 7: 68.2 vs. 61.8 mg, *p*-value: 0.049; day 14: 84.1 vs. 68.9 mg, *p*-value: 0.004; day 21: 93.5 vs. 76.4 mg, *p*-value: 0.015).

The prevalence of the six most frequent AEs according to the analgesic response and dose escalation over time is reported in Table 5. High dose escalation patients had a higher risk of experiencing an AE in responders, particularly at day 7, except for vomiting. Significant ORs were found for confusion (OR: 2.09, 95% CI: 1.18–3.71, at day 7), constipation (OR: 2.35, 95% CI: 1.39–3.98, at day 7), drowsiness (OR: 1.88, 95% CI: 1.11–3.16, at day 7 and OR: 1.76, 95% CI: 1.06–2.92, at day 14), dry mouth (OR: 1.88, 95% CI: 1.13–3.13, at day 14 and OR: 1.99, 95% CI: 1.08–3.66, at day 21), and nausea (OR: 2.29, 95% CI: 1.32–3.97, at day 14).

## 4. Discussion

The initial assumption of our study was that the assessment of the response to prolonged treatment with opioids was based on three variables: the achieved analgesia, the change of dose, and the opioid-induced AEs. Each of these variables was considered separately and then correlations between them were made. With reference to analgesia, the percentage of responders varied between 74% and 81% across the subsequent visits, and pain intensity in responders decreased by an average of 4 points. On the other hand, the percentage of non-responders ranged from 26% to 19% and pain intensity in non-responders diminished less than one point. From these data, it emerged, once again, that satisfactory analgesia was not always achieved [7,8], causing an important clinical problem especially in advance-stage cancer patients.

The daily dose of opioids on average tended to increase over time in the whole population. Low dose escalation patients were between 73% and 82% depending on the visit time. The difference in dose between low dose escalation and high dose escalation groups was quite impressive: at day 21, high dose escalation patients were prescribed a more than two-fold higher dose than low dose escalation ones. Responders received a significantly lower dose of opioids, their dose increased by an average of 50% in 21 days, and among them, the percentage of high dose escalation ranged from 16% to 22%. Conversely, non-responders were given higher opioid doses, with an increment of about 80%, and among them, high dose escalation proportion ranged from 26% to 39%. The trend was particularly critical in this last group of patients since the dose increase did not correspond to a better analgesic effect.

When we considered the prevalence of the six most common opioid-induced AEs according to the combination of opioid analgesic effect and dose escalation, we found that responders with high dose escalation experienced more frequently all AEs, except vomiting. In addition, AEs were significantly more frequent, especially at the beginning of treatment (at days 7 or 14). No substantial differences in the prevalence of AEs were observed over time in non-responders, regardless of dose increase. 

The results that emerged in this analysis can provide useful indications for the clinical evaluations and therapeutic decisions for cancer patients with pain. First, in each patient, pain intensity should be measured at each visit, and the analgesic response should be considered positive if pain decreases by at least 30% compared to the baseline value [6]. Moreover, a satisfying result for the patient corresponds to the achievement of a pain intensity absolute value of 4 or more points [23]. The application of this double criterion allows us to know the level of achieved analgesia.

Second, the dose increase over time should also be measured. The opioid dose is often increased due to the failure of the analgesic effect. It is necessary to understand to what extent the increase can be considered normal. The cut-off between low dose escalation and high dose escalation is a daily increase of 5%. Although this value has been empirically calculated [22], it remains a method commonly used and its calculation can be done using a simple formula. Attention should be paid particularly to high dose escalation patients, who are in a worrying situation that suggests a likely onset of tolerance. More rarely, opioids induce hyperalgesia, which is normally associated with high doses and an increase in pain. In these cases, the continued administration of opioids is counterproductive, while the use of other drugs, such as ketamine, is a more appropriate choice [24]. Less frequently, high opioid doses are requested due to mechanisms generating pain as, for instance, the presence of neuropathic pain, or the involvement of central neuro-inflammation, sometimes observed in chronic pain [25]. In the case of high dose escalation patients, the best-known solution is to switch the opioid with another one [26]. Alternatively, several studies have suggested the use of low-dose methadone as an add-on to regular background opioid treatment, when this is not effective despite increasing doses [27,28]. Important pain relief was reached in about 70% of 410 cancer patients without significant modifications of AEs [28]. 

Third, AEs and safety profiles should be assessed along all the care path. A simple tool, like the Edmonton Symptom Assessment System [29], based on a list of symptoms/AEs measurable by a numeric rating scale, allows us to recognize the presence of symptoms, also giving a quantitative measure of their severity. The presence and severity of symptoms, however, must be considered after taking into consideration analgesia and dose. Intuitively, we may think that the higher the dose the more frequent and severe are the AEs. Indeed, our results suggest that things go differently: the dose affects the presence of undesirable effects only in non-responders. In these patients, it seems that the opioids do not really bind to their receptors, including both analgesic receptors and those producing AEs. Any additional dose increase is unnecessary in this situation, and the only possible solution is the change in the opioid. The situation is different in responders where effective analgesia, obtained at increasing doses, is also accompanied by more frequent undesired effects. In this case, the price to pay for maintaining analgesia can be heavy. 

Fourth, a “time factor” should be considered. The response to opioid treatment is a dynamic and evolving process. Analgesia, dose, and tolerability change over time, differently in each patient. The speed of these changes is important and depends on the disease, the duration of treatment, and the prognosis. For example, a rapid dose increase can be accepted in a terminal patient where the primary goal is reducing suffering, but it becomes problematic when the disease is chronic, not advanced, and requires intercurrent cancer treatments other than long-term opioid treatment. In the latter case, frequent changes in a short time can be critical and require rapid and competent evaluation and decision-making processes. Possible solutions include the dose reduction of the opioid if analgesia does not worsen, or the co-treatment with non-opioid analgesic drugs, or, alternatively, an opioid switch [26].

The main strength of this study consists of the original idea of considering the response to opioids not only in terms of the obtained reduction in pain but also as the combination of various aspects relevant in determining the outcome and duration of the therapy. Previous studies that evaluated opioid treatment outcomes showed only a measure of positive responses, based on pain reduction by ≥30% [7]. Also, in a recent meta-analysis examining the risk factors for clinical response to opioids, the analgesic effect was the only parameter evaluated [30]. The second strength of this study is the large sample of patients with a homogeneous stage of disease. Among the limitations of our study, there is the quite-short period of observation (21 days), which can provide only an indication of the changes and interactions of the studied aspects. A more extended period of evaluation would have allowed a better understanding of the role played by the examined aspects in the long-term. Moreover, some patients dropped-off at day 21, with some difference in responders and non-responders (29 out of 329, 8.8%, in responders; and 19 out of 116, 16.4% in non-responders). Among responders, 62% died, 17% had a treatment withdrawal, 14% were transferred to other centres, and 2% dropped-out for other reasons; corresponding values in non-responders were 53%, 11%, 26% and 11%).

## 5. Conclusions

This analysis indicates that the clinical response to opioids results from the combination of three clinical aspects (the achieved analgesia, the change of dose, and the opioid-induced AEs), which are strongly interrelated, plus the “time factor”. The time factor should be evaluated in pharmacological and clinical terms. Some changes in the opioid response are related to the adaptive behavior of the drug used, such as the onset of tolerance or opioid-induced hyperalgesia. In other cases, the variations of cancer pain and its multimorphic nature in the course of the disease influence the response [9]. Consequently, the clinician should evaluate all these clinical variables together at each patient visit. A balance between opioid benefits and harms is necessary. It ranges from a full positive balance of the three aspects, where treatment can continue with efficacy and safety, to a complete negative balance, which must lead to a drastic and rapid change of therapy. Intermediate situations require targeted interventions on the negative aspect. 

## Figures and Tables

**Table 1:** Demographics and main clinical characteristics of 498 cancer patients at baseline.

	Cancer Patients (%)
Age (years), mean (SD)	66.9 (11.8)
Female	221 (44.4)
Primary site of tumour	
Lung/respiratory system	141 (28.3)
Breast	65 (13.1)
Genitourinary/Female reproductive system	65 (13.1)
Colon, rectum	57 (11.5)
Head and neck	42 (8.4)
Pancreas	39 (7.8)
Prostate	29 (5.8)
Stomach/Oesophagus	18 (3.6)
Other	42 (8.4)
Presence of metastasis	424 (85.1)
Ongoing anticancer therapy	191 (38.4)
Pain	
Average pain, mean (SD)	6.0 (1.4)
Worst pain, mean (SD)	8.0 (1.5)
Type of pain	
Nociceptive	412 (82.7)
Nociceptive /Neuropathic	80 (16.1)
Insufficient information to classify	6 (1.2)
Karnofsky Performance Status, mean (SD)	66.9 (17.0)
≤ 40	57 (11.5)
41–70	273 (54.8)
≥ 71	168 (33.7)
Concomitant diseases*	320 (64.3)
Therapies for concomitant disease°	294 (59.0)
Therapies for symptoms	187 (37.6)

SD: standard deviation. * Including metabolic/hormonal, cardiovascular, neurological/psychological, digestive system, and respiratory diseases. ° Including cardiovascular, antidiabetic, gastrointestinal, antibiotics, central nervous system, hormonal and respiratory drugs.

**Table 2:** Average pain intensity and pain intensity difference over time overall and by analgesic response among 498 cancer patients.

	Baseline	Day 7	Day 14	Day 21
All patients				
*N*	498	445	416	397
Average pain intensity (SD)	6.0 (1.36)	2.9 (1.9)	2.7 (1.9)	2.4 (1.8)
Pain intensity difference^§^ (SD)	-	−3.1 (2.2)	−3.3 (2.4)	−3.6 (2.2)
		*P*-value for trend = 0.002*		
Responders *N* (%)	−	329 (73.9)	323 (77.6)	323 (81.4)
Average pain intensity (SD)	−	2.1 (1.4)	2.0 (1.4)	1.9 (1.3)
Pain intensity difference^§^ (SD)	−	−4.0 (1.7)	−4.1 (1.7)	−4.2 (1.8)
		*P*-value for trend = 0.063*		
Non-responders *N* (%)	−	116 (26.1)	93 (22.4)	74 (18.6)
Average pain intensity (SD)	−	5.0 (1.6)	5.1 (1.4)	4.9 (1.6)
Pain intensity difference^§^ (SD)	−	−0.8 (1.5)	−0.7 (1.2)	−0.9 (1.2)
		*P*-value for trend = 0.575*		
*P*-value°		<0.001	<0.001	<0.001

SD: standard deviation. ^§^ Compared to baseline. * A linear regression model was used to detect the trend in pain intensity difference values over time. The *t*-test was performed to compare PID values between responders and non-responders.

**Table 3:** Opioid daily dose prescribed over time overall and by dose escalation among 498 cancer patients.

	Baseline	Day 7	Day 14	Day 21
All patients				
*N*	498	445	413	391
Mean Dose (mg; SD)	51.9 (16.2)	63.4 (30.3)	72.3 (44.9)	79.6 (54.5)
		*P*-value for trend <0.001*		
Low dose escalation				
N (%)	−	326 (73.3)	301 (72.9)	322 (82.4)
Mean Dose (mg; SD)	−	52.3 (16.4)	56.8 (20.29)	65.3 (28.6)
		*P*-value for trend <0.001*		
High dose escalation				
N (%)	−	119 (26.7)	112 (27.1)	69 (17.6)
Mean Dose (mg; SD)	−	94.1 (37.7)	113.9 (63.1)	146.7 (87.3)
		*P*-value for trend <0.001*		
*P*-value °		<0.001	<0.001	<0.001

SD: standard deviation. *A linear regression model was used to detect the trend for dosage over time. ° The *t*-test was performed to compare dosage values between low and high dose escalation. Note: at day 14, dosage was missing for three patients; at day 21, dosage was missing for six patients.

**Table 4:** Dose escalation according to analgesic response over time among 498 cancer patients.

	Day 7	Day 14	Day 21
**Responders**	329	321	318
Mean dose (mg; SD)	61.8 (29.3)	68.9 (37.0)	76.4 (48.1)
	*P*-value for trend <0.001*		
High dose escalation patients (%)	74 (22.5)	81 (25.2)	50 (15.7)
**Non-responders**	116	92	73
Mean dose (mg; SD)	68.2 (32.7)	84.1 (64.5)	93.5 (75.2)
	*P*-value for trend = 0.002*		
High dose escalation patients (%)	45 (38.8)	31 (33.7)	19 (26.0)
*P*-value °	0.049	0.004	0.015

SD: standard deviation. *A linear regression model was used to detect the trend for dosage values over time. ° The *t*-test was performed to compare dosage values between responders and non-responders.

**Table 5:** Adverse events over time according to analgesic response and dose escalation among 498 cancer patients.

	Day	Analgesic Response	High Dose Escalation (Events/Total)	Low Dose Escalation (Events/Total)	Odds Ratio (95% CI) *	*P*-value for heterogeneity^#^
Confusion	7	Responders	25/74	50/255	2.09 (1.18–3.71)°	0.074
		Non-responders	11/45	20/71	0.83 (0.35–1.94)	
	14	Responders	20/81	42/240	1.55 (0.84–2.83)	0.285
		Non-responders	7/31	5/61	3.27 (0.94–11.33)	
	21	Responders	9/50	38/268	1.33 (0.60–2.95)	0.947
		Non-responders	5/19	11/54	1.40 (0.41–4.72)	
Constipation	7	Responders	44/74	98/255	2.35 (1.39–3.98)°	0.036
		Non-responders	24/45	40/71	0.89 (0.42–1.88)	
	14	Responders	47/81	110/240	1.63 (0.98–2.72)	0.522
		Non-responders	16/31	29/61	1.18 (0.50–2.80)	
	21	Responders	27/50	125/268	1.34 (0.73–2.46)	0.389
		Non-responders	13/19	26/54	2.33 (0.77–7.04)	
Drowsiness	7	Responders	39/74	95/255	1.88 (1.11–3.16)°	0.446
		Non-responders	24/45	33/71	1.32 (0.62–2.78)	
	14	Responders	43/81	94/240	1.76 (1.06–2.92)°	0.774
		Non-responders	12/31	18/61	1.51 (0.61–3.74)	
	21	Responders	22/50	95/268	1.43 (0.78–2.64)	0.554
		Non-responders	8/19	23/54	0.98 (0.34–2.83)	
Dry mouth	7	Responders	30/74	83/255	1.41 (0.83–2.41)	0.723
		Non-responders	21/45	30/71	1.20 (0.56–2.54)	
	14	Responders	40/81	82/240	1.88 (1.13–3.13)°	0.461
		Non-responders	13/31	22/61	1.28 (0.53–3.10)	
	21	Responders	24/50	85/268	1.99 (1.08–3.66)°	0.039
		Non-responders	6/19	25/54	0.54 (0.18–1.62)	
Nausea	7	Responders	26/74	60/255	1.76 (1.01–3.08)°	0.689
		Non-responders	17/45	21/71	1.45 (0.66–3.18)	
	14	Responders	30/81	49/240	2.29 (1.32–3.97)°	0.068
		Non-responders	9/31	20/61	0.84 (0.33–2.15)	
	21	Responders	9/50	59/268	0.78 (0.36–1.69)	0.209
		Non-responders	9/19	18/54	1.80 (0.62–5.21)	
Vomiting	7	Responders	4/74	22/255	0.61 (0.20–1.82)	0.179
		Non-responders	8/45	8/71	1.70 (0.59–4.92)	
	14	Responders	8/81	23/240	1.03 (0.44–2.41)	0.180
		Non-responders	2/31	11/61	0.31 (0.06–1.51)	
	21	Responders	3/50	24/268	0.65 (0.19–2.24)	0.659
		Non-responders	2/19	12/54	0.41 (0.08–2.04)	

CI: confidence interval. * Odds ratio (OR) for high dose escalation versus low dose escalation, estimated from a logistic regression model. ° *P*-value < 0.05. ^#^The Breslow–Day test was used to detect heterogeneity of OR estimates between responders and non-responders.

## Abbreviations

AE: adverse events; API: Average Pain Intensity; OEI: Opioid Escalation Index; OMEDD: oral morphine-equivalent daily dose; OR: odds ratio; PID: pain intensity difference; SD: standard deviation; WHO: World Health Organization.
